# Circulatory collapse during wound closure in spine surgery with an unknown cause: a possible adverse effect of topical application of vancomycin?

**DOI:** 10.1186/s12871-020-01220-6

**Published:** 2021-01-06

**Authors:** Xiaoqing Zhang, Wenwen Zhai, Min Li, Xiangyang Guo

**Affiliations:** Department of Anesthesiology, Peking University Third Hospital, No. # 49, Huayuan Rd; Haidian Dist, Beijing, China

**Keywords:** Vancomycin, Hypotension, Shock, Anaphylaxis

## Abstract

**Background:**

Vancomycin (VCM) is effective in fighting Gram-positive bacteria related severe infections, and topical application of VCM powder is widely used in orthopedic surgery to prevent wound infection. However, VCM could lead to infusion rate-dependent antibody-and complement-independent anaphylaxis reaction by inducing direct release of histamine.

**Case presentation:**

We retrospectively analyzed seven cases of severe hypotension and shock during wound closure or immediately after orthopedic surgery with unidentifiable reasons. We found that these cases were all associated with local application of VCM powder during wound closure process. Two patients experienced sudden cardiac arrest. Most of the cases (6/7) with circulatory collapse were discharged without severe sequelae. While one case with application of 3 g VCM developed cardiac arrest and remained in a coma due to hypoxic-hypoxic encephalopathy. The clinical presentations and the time of the shock onset were considered to be related with a VCM induced anaphylaxis reaction. However, as this was a retrospective study, and there was no laboratory examination performed, the conclusion was made upon differential diagnosis based on clinical manifestations and the timing of the shock.

**Conclusions:**

Local application of VCM may not be as safe as was once believed and may lead to a related anaphylaxis. As VCM induced infusion-rate dependent, non-IgE mediated anaphylaxis is characterized by delayed occurrence, severe hypotension and even circulatory collapse, surgeons and anesthesiologists should be extra vigilant during and after VCM application.

## Background

Shock is defined as a state of insufficient perfusion and oxygen delivery to the tissues. The pathophysiology of shock could be simplified to three major components: cardiac function (the pump), intravascular volume (the tank), and systemic vascular resistance (the pipes) [1]. Whatever the causes of shock may be, if the impaired perfusion and oxygen delivery is not recognized and reversed, the circulatory collapse may progress into organ dysfunction and failure, tissue necrosis and even death. It is urgent to identify the causes of shock and take immediate measures. However, in critical situations, it is hard to rapidly distinguish the culprit during complex clinical situations. Here we present 7 cases (Table 1) of unidentifiable causes of shock during wound closure or shortly after spine surgery, and discuss the possible reasons.

**Table 1:** Summary of clinical presentations of seven patients with circulatory collapse and unknown etiologies during or shortly after wound closure in spine surgery

Case	Demographic information	Initial signs/symptoms of shock and management	Hemoglobin value (g/L), fluid resuscitation and blood product infusion	VCM administration and Postoperative sequelae
1	Female; 68-yr; 160 cm/60 kg BMI: 23.4 Diagnosis: Lumbar stenosis (L3-S1) Surgery: Posterior decompression, fixation and fusion of lumbar spine. Medical and allergy history: Hypertension History of retina surgery	Time of occurrence: Subcutaneous layer closure, 30 min after initiation of wound closure. Signs and symptoms: ABP: 30/20 mmHg SpO_2_: undetectable ECG: tarchycardia with elevated ST segment Skin and airway symptoms: none. Management: Boluses and continuous infusion of noradrenaline (0.01–0.04 μg/kg/min) for 45 min. Fluid resuscitation and blood transfusion.	Preoperative HGB value: 124 Intraoperative minimum value of HGB: 73 Blood loss: 600 ml Urine: 850 ml Fluid and blood product infusion: Crystalloid: 3450 ml Colloid: 500 ml PRBC infusion: 1200 ml Infused autologous blood: 210 ml	VCM administration: 0.5 g mixed with bone debris into the cage and 0.5 g spraying on the dura mater and into the mascularis layer. Postoperative sequelae: Proceeded with surgery and the patient was extubated uneventfully.
2	Male; 74-yr; 171 cm/67 kg BMI: 23.9 Diagnosis: lumbar stenosis (L3–5) Surgery: Posterior decompression, fixation and fusion of lumbar spine. Medical and allergy history: nil relevant.	Time of occurrence: Subcutaneous layer closure, 30 min after initiation of wound closure. Signs and symptoms: NBP: 45/15 mmHg SpO_2_: undetectable ECG: tachycardia with ST segment depression Skin and airway symptoms: none. Management: Boluses and continuous infusion of noradrenaline (0.05–0.4 μg/kg/min) and adrenaline (0.5–0.15 μg/kg/min) for 4 h.	Preoperative HGB value:135 Intraoperative minimum value of HGB: 79 Blood loss: 600 ml Urine: 1950 ml Fluid and blood product infusion: Crystalloid: 6300 ml Colloid: 500 ml PRBC infusion: 1200 ml Plasma: 800 ml Infused autologous blood: 254 ml	VCM administration: 0.5 g mixed with bone debris into the cage and 0.5 g spraying on the dura mater and into the mascularis layer. Postoperative sequelae: The patient was extubated and transferred to ICU due to neurological symptoms (unable to follow instructions). Postoperative imaging: intracranial minor hemorrhage at subdural and subarachnoid space. The patient was discharged from hospital without neurological deficit.
3	Male; 63-yr; 169 cm/70 kg BMI: 24.5 Diagnosis: Lumbar stenosis (L3–5) Surgery: Posterior decompression, fixation and fusion of lumbar spine. Medical and allergy history: Lacunar infarction; Bilateral carotid atherosclerotic plaque formation	Time of occurrence: 50 min after initiation of wound closure and 20 min post-extubation in the PACU. Signs and symptoms: NBP: 60/25 mmHg SpO_2_: 96% HR:sudden elevation from 60 to 90 bpm Postoperative agitation Skin and airway symptoms: none. Management: Boluses of ephedrine, phenylephrine and fluid resuscitation.	Preoperative HGB value: 176 Intraoperative minimum value of HGB:123 Blood loss: 800 ml Urine: 800 ml Fluid and blood product infusion: Crystalloid: 2350 ml Colloid: 1000 ml PRBC infusion: 400 ml	VCM administration: Topical spraying of vancomycin (1 g) on the dura mater and into the mascularis. Postoperative sequelae: Uneventful.
4	Female; 70-yr; 160 cm/65 kg BMI:25.4 Diagnosis: Lumbar stenosis (L4-S1) Surgery: Posterior decompression, fixation and fusion of lumbar spine. Hypertension Diabetes Mellitus	Time of occurrence: Subcutaneous layer closure, 30 min after initiation of wound closure. Signs and symptoms: NBP: 45/15 mmHg SpO_2_: undetectable Persistent tachycardia Skin and airway symptoms: none. Management: Boluses of phenylephrine for treating severe hypotension and esmolol for tachycardia Fluid resuscitation and blood transfusion.	Preoperative HGB value: 150 Intraoperative minimum value of HGB: 92 Blood loss: 1500 ml Urine: 1800 ml Fluid and blood product infusion: Crystalloid: 3100 ml Colloid: 1500 ml PRBC infusion: 1200 ml Infused autologous blood: 450 ml	VCM administration: 0.5 g mixed with bone debris into the cage and 0.5 g spraying on the dura mater and into the mascularis layer. Postoperative sequelae: The patient was extubated after surgery and transferred to ICU due to tachycardia after sufficient fluid resuscitation. Recovered uneventfully.
5	Female;48-yr; 170 cm/75 kg BMI: 26.0 Diagnosis: Thoracic spinal canal stenosis (T6-T11) Surgery: Posterior decompression, fixation and fusion of thoracic spine. Medical and allergy history: nil relevant.	Time of occurrence: Subcutaneous layer closure, 30 min after initiation of wound closure. Signs and symptoms: NBP: 40/30 mmHg to undetectable HR: 120 bpm to 40 bpm SpO_2_: undetectable Cardiac arrest Skin and airway symptoms: none. Management: Extracardiac compression Boluses of noradrenaline (200 μg) and adrenaline (1 g) Continuous infusion of noradrenaline (0.02–0.08 μg/kg/min) for 50 min Fluid resuscitation and blood transfusion.	Preoperative HGB value: 132 Intraoperative minimum value of HGB: 74 Blood loss: 2500 ml Urine: 1300 ml Fluid and blood product infusion: Crystalloid: 4000 ml Colloid: 500 ml PRBC infusion: 2400 ml	VCM administration: Topical spraying of vancomycin (1 g) on the dura mater and into the mascularis layer. Postoperative sequelae: The patient was extubated after surgery, and discharged from hospital uneventfully.
6	Male; 66-yr; 175 cm/ 80 kg BMI: 26.1 Diagnosis: Lumbar stenosis (L3–5) Surgery: Posterior decompression, fixation and fusion of lumbar spine. Medical and allergy history: Carotid artery stenosis Diabetes Mellitus	Time of occurrence: Sudden cardiac arrest 10 min after extubation (approximately 45 min after initiation of wound closure) with full recovery from anesthesia and without any discomfort complaint. Signs and symptoms: Cardiac arrest and persistent VF Skin and airway symptoms: none. Management: Continuous CPR Persistent VF was treated with repeated defibrillation Boluses of adrenaline (a total of 6 mg) and amiodarone. Fluid resuscitation and blood transfusion.	Preoperative HGB value: 143 Intraoperative minimum value of HGB: 78 Blood loss: 1000 ml Urine: 850 ml Fluid and blood product infusion: Crystalloid: 4400 ml Colloid: 1000 ml PRBC infusion: 1200 ml Infused autologous blood: 600 ml	VCM administration: Topical spraying of vancomycin (3 g) on the dura mater and into the mascularis. Postoperative sequelae: The patient was reintubated and transferred to ICU after ROSC. The patient was diagnosed as hypoxic encephalopathy and remained in a coma state. Tracheotomy was performed 2 weeks after surgery, and the patient was transferred to a nursing home for rehabilitation 1 month after orthopedic surgery.
7	Female; 43-yr; 155 cm / 54 kg BMI: 22.4 Diagnosis: Thoracic kyphosis (T7-T8) Surgery: Osteotomy for kyphosis of thoracic spine (T7-T8). Medical and allergy history: nil relevant.	Time of occurrence: subcutaneous layer closure, 30 min after initiation of wound closure. Signs and symptoms: HR:tachycardia NBP: 60/30 mmHg SpO_2_ 82% Skin and airway symptoms: none. Management: Boluses of phenylephrine and ephedrine; Continuous infusion of noradrenaline (0.05–0.1 μg/kg/min) Fluid resuscitation and blood transfusion.	Preoperative HGB value: 138 Intraoperative minimum value of HGB: 95 Blood loss: 1200 ml Urine: 1900 ml Fluid and blood product infusion: Crystalloid: 4700 ml Colloid: 1000 ml PRBC infusion: 1200 ml Infused autologous blood: 470 ml	VCM administration: Topical spraying of vancomycin (1 g) on the dura mater and into the mascularis layer. Postoperative sequelae: The patient was transferred to ICU for optimal monitoring and recovered uneventfully.

Abbreviations: *NBP* Non-invasive blood pressure; *ABP* Arterial blood pressure; (NBP is noted when ABP is not available.) *VF* Ventricular fibrillation; *CPR* Cardiopulmonary Resuscitation; *HGB* Hemoglobin; *VCM* Vancomycin; *PRBC* Packed red blood cell; *ROSC* Return of spontaneous circulation

## Case presentation

The first case we encountered was a 68-yr female with past medical history of hypertension and surgical treated retina detachment, who underwent posterior decompression, fixation and fusion of lumbar spine due to lumbar stenosis (L3-S1). The operation was uneventful until sudden circulatory collapse occurred during the process of rinsing mascularis and superficial fascia layer whilst closing the wound. A sudden drop in arterial blood pressure (ABP) (130/60 mmHg to 30/20 mmHg), increased HR (60 bpm to 100 bpm) with elevated ST-segment and significant decrease of PetCO_2_ (31 mmHg to 16 mmHg) were noticed, with undetectable pulse oximetry read. The patient was turned to supine position immediately and managed with boluses (40–100 μg) and a continuous infusion of noradrenaline (0.01–0.04 μg/kg/min) for approximately 45 min. The circulation was gradually stabilized. No rash, erythema, or bronchospasm were noted. The bilateral breath sounds were equal and the airway pressure remained normal. We continued treatments of fluid resuscitation and blood transfusion with corticosteroid and ice cap to prevent hypoxic-ischemic encephalopathy. Intraoperative emergency consultations and immediate examinations of 12-lead ECG, point-of-care transthoracic echocardiography (TTE), and full panel laboratory tests were performed. However, these results did not support the causes of sudden cardiac dysfunction (the pump) including mechanical obstruction (pericardial tamponade and massive pulmonary embolus), acute myocardial infarction, acute valvular insufficiency, and arrhythmia. Vascular catastrophes were excluded and there were no signs of sepsis. We also examined the intravascular volume function (the tank) by performing the TTE, and ruled out intraperitoneal major hemorrhage which we once encountered (caused by inadvertent piercing of iliac artery when placing probe during lumbar surgery) by examination of abdominal ultrasound and hemoglobin level; Volume status was evaluated by measuring CVP, urine output, infused fluid volume; Surgical blood loss was reviewed to exclude hemorrhage shock and hypovolemia. As no special medications were given except anesthetic maintenance drugs, and no signs of mottled skin or bronchospasm were noticed, anaphylaxis was not considered at that time. After restoring circulatory stability, the surgery preceded uneventfully. The patient was discharged from hospital 7 days later.

It was intriguing that within 1 month, another case of circulatory collapse happened during wound closure in spine surgery performed by the same orthopedic surgeon. The intraoperative clinical manifestations were very similar to the previous one. Hemodynamic resuscitation were initiated immediately with repeated boluses and continuous infusion of noradrenaline (0.05–0.4 μg/kg/min) and adrenaline (0.5–0.15 μg/kg/min). After stabilizing the circulation 4 h later, the patient was extubated and transferred to ICU due to neurological symptoms (unable to follow instructions after full recovery from anesthesia). Postoperative imaging revealed intracranial micro hemorrhagic foci in the subdural and subarachnoid space. The patient was discharged from hospital without any neurological sequelae.

With further exploration and analysis of the similarities of these two cases, we found that local application of vancomycin (VCM) powder was used in these two patients to prevent postoperative surgical site infections (SSIs) during wound closure (0.5 g VCM loaded with bone debris into the cage and another 0.5 g sprayed on the dura mater and into the mascularis). Ramamani Mariappan reported a case of circulatory collapse after topical application of vancomycin powder during spine surgery, which exhibited almost the same clinical course as ours [2]. It was speculated that the rapid absorption of VCM applied to the surgical wound caused an anaphylaxis reaction and circulatory collapse. The reaction occurred approximately 30 min after the application and persisted for 6 h. The serum tryptase level was not elevated, suggesting that VCM caused a direct histamine release, which is in keeping with our current understanding. A similar case reported a 74-year-old woman who underwent a primary total knee replacement presented with red man syndrome in the PACU 45 min after release of tourniquet following the use of VCM-loaded bone cement. The patient remained fully conscious despite a sudden drop in blood pressure to 47/34 mmHg [3]. Therefore, local application of VCM may pose the risk of causing severe circulatory adverse effect.

Similar circulatory collapse cases in spine surgery without definitive causes during wound closure or shortly after surgery, though rare, were encountered in our institution. Therefore we retrospectively analyzed these cases for the past 2 years, and asked the anesthesiologists and orthopedic surgeons in charge for further information. A total of 7 cases were collected (Table 1). There were 3 males and 4 females, the median age was 66 y (43–74 y), and the averaged BMI was 24.39 ± 1.51 (22.3–26.1). It was interesting to note that these cases were all associated with topical application of VCM powder (Table 1). In these 7 cases, the onset time of severe circulatory fluctuation was 30 min in 5 cases, 50 min in one case (applied with 1 g of VCM), and approximately 45 min in another case (locally applied 3 g of VCM), respectively. Most of the cases (6/7) with severe circulatory fluctuation were corrected without severe sequelae. While in one case in which 3 g of VCM was applied on the dura mater and into the mascularis during wound closure, sudden cardiac arrest happened shortly after the patient had fully recovered from the anesthetic, following surgery and extubation in the OR. CPR was initiated immediately. The patient was reintubated and ventilated mechanically. The persistent VF was treated with continuous chest compressions and repeated defibrillation, with boluses of adrenaline (a total of 6 mg) and amiodarone. After 50 min of continuous CPR, with fluid resuscitation and blood transfusion, the patient was transferred to ICU following the return of spontaneous circulation and reverting into sinus rhythm. However, the patient remained in a comatose state due to hypoxic-hypoxic encephalopathy, and tracheotomy was performed 2 weeks later.

## Discussion and conclusion

### Vancomycin and anaphylaxis reaction

“International Consensus on (ICON) Anaphylaxis” define anaphylaxis as “a serious, generalized or systemic, allergic or hypersensitivity reaction that can be life threatening or fatal” [4]. It has been demonstrated that there exists IgE-dependent and three IgE-independent mechanisms in anaphylaxis pathophysiology [5]. VCM could directly activate mast cells to release histamine and other mediators through an IgE-independent mechanism that is calcium-, phospholipase C–, and phospholipase A2–dependent but otherwise unknown [5, 6]. Vancomycin-induced direct histamine release is also infusion rate dependent [7]. This IgE-independent anaphylaxis reaction generally disappears within 20 mins, but may linger for hours. The most common manifestation is a sudden drop in blood pressure, which is related to the negative inotropic effect and the vasodilator action stimulated by the histamine release. Alteration in vascular tone is also a common feature of anaphylactic shock [8]. Richter J and colleagues demonstrated in rats that VCM could directly influence the vascular tonus, affecting the microcirculation [9].

The VCM reactions studied in the pediatric population revealed that non-IgE mediated reaction accounts for the vast majority of reactions (92%; prevalence, 5.4–5.8%), whereas possible IgE-mediated vancomycin reactions were exceedingly rare (prevalence, 0.0–0.37%) [7]. For adults, the reported incidence of non-IgE mediated reaction varies between 3.7 and 47% in infected patients and 90% in healthy volunteers [2]. Elevated serum mast cell tryptase is a valuable indicator for diagnosing Ig-E mediated anaphylactic reaction. However, it is not useful in determining a non-IgE mediated anaphylactic reaction caused by histamine release [2]. Detection of histamine concentration in clinical blood specimens is difficult due to its extremely short half-life, and histamine is not a mast cell specific product [10]. As this study is retrospective in nature, and our hospital does not perform examination of serum tryptase and histamine concentration, we could not differentiate whether the adverse effects were caused by VCM induced IgE-mediated or non-IgE mediated reaction.

### Topical application of vancomycin powder and surgical site infections

VCM is effective in fighting Gram-positive bacteria related severe infections [11]. While the actual utility of local application of VCM is controversial. After the first reported topical application of VCM by orthopedic surgeons to reduce postoperative surgical site infections (SSIs) rate [12], a large number of meta-analyses, retrospective cohort studies, and randomized controlled trials were published to assess the power of this method in preventing SSIs. Recent findings from large-scale propensity score matched analyses, meta analyses, RCTs studies, and observational studies of spine surgeries and knee arthroplasties all demonstrated that the intrawound VCM powder application may not decrease SSIs, but may increase postoperative complications [13–19].

VCM is an antibiotic that was considered safe in local application and has been widely used in hospitals. However, dose recommendations, dilutions, monitoring, infusion types and rates are still controversial [20]. There’s no defined time or situation during surgery when local VCM should be applied. Some may load VCM powder with bone debris into the cage, or apply VCM powder directly onto the dura mater or after closure of the fascia, whereas other studies reported that VCM powder was applied in the subfascial layer [2, 13]. However, intrawound absorption rate of VCM powder is uncontrollable as this depends on multiple factors, such as the extent and degree of tissue/muscle damaged by surgical manipulation, anatomic location where VCM powder is placed (mixed with bone debris, or between muscularis layers that are abundant with blood supply), and the surgeon’s suturing maneuvers (the flushing saline would leak from upper layer into the layer where VCM powder is deposited). These factors all pose potential risk of causing rapid drug dissolution and accelerated absorption into the circulatory system. Moreover, individual’s adverse reactions to VCM varied. These incidental events may be accidentally linked and lead to severe outcome. Since intravenous administration of VCM could lead to adverse effects, it is theoretically possible that local application of VCM powder may cause similar clinical manifestation.

Topical application of VCM was intended to achieve the effect of providing high local concentrations while avoiding high systemic levels and the risk of bacterial resistance. The serum concentration level peaks at 30 min and then declines to baseline at 6 h [2]. This may explain the delayed and unpredictable occurrence of anaphylaxis reactions after local application of VCM powder, due to the uncertainty of the drug absorption rate and individual reaction differences. The instructed therapeutic dosage of VCM is 30 mg/kg/day which should be fractioned in 2 or 3 doses. Moreover, the infusion rate should be within 10 mg/min, and last for more than 60 min. For elderly patients, this dosage should be reduced [21, 22]. Although the most commonly used dose of intrawound VCM is 1 g, some authors reported that they altered the dose according to the length of the incision [23]. VCM is not the first-choice antibiotic as its adverse effects are frequently reported including hypotension and tachycardia, phlebitis, nephrotoxicity, ototoxicity, hypersensitivity reactions, red man syndrome, neutropenia, chills, fever, interstitial nephritis, etc. [13]. As VCM induced adverse reactions are dose and infusion-rate dependent, doctors should be highly vigilant to over-dosing which might bring disastrous results.

### Other issues related to shock

In this case series, the typical signs of erythema and redness (red man syndrome) have not appeared, which resemble the previous case reported by Mariappan R and colleagues [2]. The lack of skin flushing may be the result of reduced perfusion due to circulatory shock or the adverse effect of VCM mainly manifested as severe alteration of vascular tonus [8, 9]. In our experience, a rapid but low dose intravenous infusion of VCM induced anaphylaxis reaction was not necessarily associated with skin manifestations. Whether this is related to racial differences or rapid absorption of low dose VCM was not known. However, the clinical features of hypotension, tachycadia and low oxygenation were notable.

For this case series, as there is no definitive laboratory examination or gold standard to help reach a definitive diagnosis of VCM induced anaphylaxis reaction, the conclusions were made upon differential diagnosis based on clinical manifestations and the timing of the shock. Other possible causes or contributing factors for the development of severe hypotension and shock need to be considered. For example, restrictive fluid therapy during surgical procedure and restrictive blood transfusion strategy leading to hypovolemia and hypotension, complicated with unseen bleeding from surgical site and other compounding factors such as hypoproteinemia and unaltered anesthetic depth during wound closure.

Another issue needs to be addressed is that there’s a notable decrease in hemoglobin (HGB) concentration in all cases when severe hypotension occurred. However, the hemorrhagic shock induced by surgical blood loss was ruled out. We speculate that this might be due to the infusion of large amounts of fluid during resuscitation. It is reported that the administration of 500 ml of fluids may acutely decrease the HGB concentration by about 1 g/dl, or about 8%, by “dilutional anemia” [24]. In septic shock patients, approximately 30% decrease of hematocrit was commonly observed in patients receiving large amounts of fluids during resuscitation, which further indicates more blood transfusion [25]. In the current study, the HGB concentrations were all measured after the initiation of fluid resuscitation and this may at least partially explained the sharp decrease of the HGB level (Table 1). When severe hypotension with unknown causes occur, initial management is to infuse large amounts of fluid with vaso-constrictive drugs. The iatrogenic hemodilution related blood transfusion may result in dilutional coagulopathy and increase surgical bleeding. This then leads to a decreased HGB level below the acceptable transfusion threshold, leading to further blood transfusion in the absence of significant blood loss [26]. This is especially true when in the scenario of the existence of suspected hemorrhagic shock.

In conclusion, local application of VCM powder during wound closure could cause delayed occurrence of severe hypotension and shock in spine surgery. Hemodynamic changes may be under-emphasized and under-reported as in most cases, this hemodynamic reaction is easy to correct and normally wouldn’t cause serious consequences. However, the actual occurrence is not rare and this might be confused with hypovolemia or anesthetic induced hypotension. Surgeons and anesthesiologists should be highly aware of VCM induced IgE-independent anaphylaxis reactions. The differential diagnosis of anaphylactic shock must be taken into consideration in patients with acute hypotension during or immediately after wound closure. Prompt laboratory analysis of serum tryptase and histamine concentration could help differentiate the causes. Moreover, since controversies still exist in the actual effect of intrawound application of VCM powder to reduce SSIs in spine surgery, local application of this drug should be used with caution.

## Data Availability

All data generated or analyzed during this study are included in this published article. More detailed information could be find in Table 1.

## Abbreviations

VCM: Vancomycin
ABP: Arterial blood pressure
TTE: Transthoracic echocardiography
